# Persistent hiccup as an adverse effect of amisulpride in a patient with first episode of psychosis.

**DOI:** 10.1192/j.eurpsy.2023.2140

**Published:** 2023-07-19

**Authors:** I. Retsou, D. Antoniadis

**Affiliations:** 1 4th PICU, Mental Health Hospital of Thessaloniki; 2 School of Medicine, Aristotle University of Thessaloniki, Thessaloniki, Greece

## Abstract

**Introduction:**

A 28-year old male patient was admitted involuntarily to the 4^th^ PICU of the Mental Health Hospital of Thessaloniki, due to severe psychotic symptoms and disorganised behaviour. Upon mental health examination the symptoms included auditory hallucinations, tangible speech, delusional ideas of somatic and persecutory type and significant neglect of his personal hygiene. The onset of his psychotic illness was 3 years prior, with two hospitalizations in the UK, and several unsuccessful attempts of outpatient monitoring.

**Objectives:**

The study of persistent hiccup as an adverse effect of antipsychotic medication.

**Methods:**

Monitoring for adverse effects during admission.

**Results:**

On the 8^th^ day of admission, oral olanzapine 10 mg was commenced at night. On the 9^th^ day, olanzapine was increased to 20 mg per day. Discontinuation of intramuscular medication occurred on the 16^th^ day. After presenting no clinical improvement for 27 days with the administration of olanzapine as monotherapy, amisulpride at 2ml per day was initiated alongside the Olanzapine. In the context of medication titration, amisulpride reached 8ml per day, equivalent to 800mg per day after 2 months of hospitalisation.

**Conclusions:**

Apart from minor constipation, no other gastrointestinal health problems were reported in his records. The onset of the hiccups occurred along the dosages of 800mg/day of amisulpride and 20mg/day of olanzapine.

There was a satisfactory response to treatment evidenced by a 30% reduction on the Positive scale of the PANSS, however, the hiccups did not recede.

**Disclosure of Interest:**

None Declared

